# Mutagenesis Approaches and Their Role in Crop Improvement

**DOI:** 10.3390/plants8110467

**Published:** 2019-10-31

**Authors:** Juhi Chaudhary, Rupesh Deshmukh, Humira Sonah

**Affiliations:** 1 Department of Biology, Oberlin College, Oberlin, OH 44074, USA; 2 National Agri-Food Biotechnology Institute (NABI), Sector 80, SAS Nagar, Mohali, Punjab 140306, India

**Keywords:** mutagenesis, mutation sequencing, CRISPR, genome editing, proteomics, next-generation sequencing (NGS)

## Abstract

Induced mutagenesis is one of the most efficient tools that has been utilized extensively to create genetic variation as well as for identification of key regulatory genes for economically important traits toward the crop improvement. Mutations can be induced by several techniques such as physical, chemical, and insertional mutagen treatments; however, these methods are not preferred because of cost and tedious process. Nonetheless, with the advancements in next-generation sequencing (NGS) techniques, millions of mutations can be detected in a very short period of time and, therefore, considered as convenient and cost efficient. Furthermore, induced mutagenesis coupled with whole-genome sequencing has provided a robust platform for forward and reverse genetic applications. Moreover, the availability of whole-genome sequence information for large number of crops has enabled target-specific genome editing techniques as a preferable method to engineer desired mutations. The available genome editing approaches such as ZFNs (Zinc Finger Nucleases), transcription activator like effector nucleases (TALENS), and clustered regularly interspaced short palindromic repeats (CRISPR)/CRISPR-associated9 (Cas9) endonuclease have been utilized to perform site-specific mutations in several plant species. In particular, the CRISPR/Cas9 has transformed the genome editing because of its simplicity and robustness, therefore, have been utilized to enhance biotic and abiotic stress resistance. The Special Issue of *Plants* highlights the efforts by the scientific community utilizing mutagenesis techniques for the identification of novel genes toward crop improvement.

Mutation breeding and plant mutagenesis play a significant role in increasing the genetic variability for desired traits in various food crops [1,2,3]. Induced mutagenesis is one of the most efficient tools used for the identification of key regulatory genes and molecular mechanisms. It is a promising approach to develop new varieties with improved agronomic characteristics, such as higher stress tolerance potential (biotic and abiotic stress) and bio-fortification. Additionally, various mutagenesis approaches have been used to study the evolutionary relationship as well as for the genetic improvement of many organisms, including microbes, animals, and plants [4,5]. Technological advances in molecular biology have re-augmented the mutation breeding making it more efficient than ever thought before. 

Numerous induced mutagenesis methods are available for plants [6]. Over the last century, physical mutagens, like fast neutron, UV, X-ray, and gamma radiations, and chemical mutagens, including N-methyl-N-nitrosourea (MNU), sodium azide, hydrogen fluoride (HF), methyl methanesulfonate (MMS), or ethyl methanesulfonate (EMS), have been widely explored. Furthermore, biological mutagens include Agrobacterium and transposon-based chromosomal integration [7]. The EMS-induced mutation is a highly effective method and, therefore, commonly used in crop breeding to develop improved crop varieties. Ramkumar et al., 2019, tested three EMS-induced stay-green mutants and wild-type for their effectiveness for drought tolerance in rice. The study reported a novel functional stay-green mutant that displayed better harvest index under both irrigated and drought conditions which can be further utilized to develop high-yielding lines [8]. In addition, induced mutagenesis is being applied to improve medicinal plants due to their high demands. For example, *Nigella sativa* mutant lines were generated by induced mutagenesis and further characterized by proteomic analysis in order to identify the proteins which were possibly associated with the plant height, seed yield, and thymoquinone content in the mutant lines. The identified proteins are mostly involved in carbon metabolism, signaling mechanisms, the differentiation of cells, carbohydrate catabolism, and secondary metabolism; therefore, the identified proteins can be further exploited toward the improvement of medicinal plants [9].

Recently, use of fast neutron bombardment to generate a mutagenized population gaining popularity due to resulting large deletion (sometimes >1 Mb) and higher number of nonrepairable double lesions as well as chromosome rearrangement in the genome [10,11]. Kumawat et al., 2019, highlighted the utility of fast neutron mutagenesis to develop a resource of gene deletion lines [12]. Such resources will be helpful for functional genomics and also to perform reverse genomics in plants. Fast neutron mutagenesis has been successfully employed in Arabidopsis, soybean, rice, and peanut. However, developing fast neutron mutagenesis facility is more difficult than the conventional gamma radiation mutagenesis. Significant efforts are needed to identify different sources that can be efficiently used to get high-intensity fast neutron radiation. 

Furthermore, gene inactivation has been successfully employed in determining the function of unknown genes in several plant species. In this approach, sense and antisense copies of a target gene are introduced in order to inactivate the endogenous genes. In other words, a DNA sequence (T-DNA, transposon, or retrotransposon) is introduced to mutate and tag the gene [13,14]. Ram et al., 2019, elaborated the role of insertional mutagenesis approaches in rice improvement. The article summarized the available T-DNA and transposon mutant resources in rice which will accelerate functional genomics studies in rice [15].

With the advancements in next-generation sequencing (NGS) techniques, detection of millions of mutations in a short period of time has become convenient and cost-efficient. Lately, for example, high-throughput next-generation sequencing-based techniques such as MutMap, MutMap-Gap, MutChromSeq, Mut-Ren-Seq, and whole-genome sequencing-based mutation mapping have been developed. These methods have high applicability as well as cost-effective tools to identify desired genes in lesser time [16,17,18]. Chaudhary et al., 2019, reviewed novel next-generation sequencing based mutation mapping approaches which are highly crucial to accelerate the mutation breeding in tomato. The article discussed the application of induced mutagenesis as well as current progresses and challenges involved in tomato mutagenesis research [19].

Despite the fact that physical and chemical-induced mutagenesis has accelerated the crop improvement, these methods are still less preferred due to random mutation, cost, and time effectiveness. However, transgenic approaches are more tedious and also need to fulfill costly regulatory norms. Nevertheless, with the recent advents in genome editing approaches have revolutionized to precisely engineer a desired mutation in the genome; for example, TALEN (transcription activator-like effector nuclease) or ZFN (zinc finger nuclease) and the CRISPR (clustered regularly interspaced short palindrome repeats). Zinc finger nucleases (ZFNs) based genome editing is one of the primitive technologies, which makes it possible to perform precise site-specific mutations and have been used in several plant species. Hilioti et al. (2016) elaborated the effectiveness of the ZFN approach to creating a variation in a LEAFY-COTYLEDON1-LIKE4 (L1L4) gene; however, the use of ZFN has become less frequent [20]. Later on, TALEN technology based on sequence-specific nucleases became a powerful tool for genome editing and have been exploited by a huge number of approaches in many different organisms [21]. Until recently, ZFNs and TALENs became least preferred choice after the development of ground-breaking genome editing tool like CRISPR/Cas9-based genome editing. This technique involves RNA-guided engineered nucleases and has been successfully applied in many models and crop plants because of its simplicity, efficiency, and versatility [22].

Overall, lots of efforts are being initiated to integrate the mutagenesis, PCR-based methods, and mapping techniques (NGS techniques) to explore the function of key genes further. The precise genome-editing tools have significantly facilitated the creation of mutant populations for economically important traits. It would be fascinating to explore the integrative network/platforms to boost functional genomics discoveries.
